# Conditions and strategies high school students living in lodgings experience as important and helpful for their well-being

**DOI:** 10.1080/17482631.2018.1481310

**Published:** 2018-06-18

**Authors:** Wenche Wannebo, Siri Andreassen Devik, Lisbeth Uhrenfeldt

**Affiliations:** a Faculty of Nursing and Health Sciences, Nord University, Bodø, Norway; b Centre for Care Research Mid-Norway, Namsos, Norway

**Keywords:** Adolescents, lodgings, senior high school, well-being, transition

## Abstract

Many 15- to 16-year-old adolescents in Scandinavian countries have to move away from home to live in lodgings during senior high school. This transition might affect the adolescents’ well-being and mental health, with the risk of dropping out of school and/or potential mental health problems. This study aims to explore what adolescents experience to be of importance and helpful for their well-being through their lives in lodgings during senior high school. Qualitative, in-depth interviews were performed among 21 adolescents from the ages of 16 to 18 of both genders living in lodgings in a Norwegian county. Eleven interviews were performed in 2008 and 10 interviews performed in 2016. Interviews were analysed using qualitative content analysis. Two main categories and six subcategories were identified as important for well-being: (1) Conditions for well-being, me and my surroundings: (a) practical support; (b) relational support; (c) convenient housing; and (d) supportive class environment. (2) Strategies for well-being, me and myself: (a) practical strategies, and (b) strategies facing challenges. Adolescents living in lodgings experience several conditions in their surroundings to be of importance for their well-being, based on which they develop their own strategies to feel well. Implications towards promoting and strengthen their well-being are discussed.

## Introduction

In some Western countries, adolescents from the age of 15–16 leave junior high school to attend senior high school. Transitions to new schools are considered a stressful event (Barber & Olsen, 2004), and this transition may be related to changes in health and well-being among adolescents (Seidman & Allen, 1994; Symonds, Dietrich, Chow, & Salmela-Aro, 2016). Changes in the adolescents’ environment include such experiences as lessened contact with close friends (Darmody, 2008), changes in the structure of schooldays, downsized contact with teachers (Darmody, 2008; Pereira & Pooley, 2007), reduced support structures (Smyth, McCoy, & Darmody, 2004) and increased academic demands (Myklebust, 2002; Pereira & Pooley, 2007).

Adolescents in Scandinavian countries, with an estimate of one out of every five, have to leave their family home at the age of 15–16 to live in lodgings during senior high school (Statistics Norway, personal communication, 15 September 2016; Statistics Sweden, personal communication, 15 September 2016; Statistics Finland, personal communication, 16 September 2016). Leaving home at an early age entails different kinds of transitions, and moving away from home at this age, while simultaneously attending a new school, seems to affect adolescents’ well-being, creating the risk of dropping out of school and/or potential mental health problems (Markussen, 2005, 2011). An increase in mental health problems in adolescents, especially among girls, during the last 20 years has been identified (Bor, Dean, Najman, & Hayatbakhsh, 2014; Collishaw, 2015; Von Soest & Wichstrøm, 2014). Likewise, a Norwegian study found that students living in lodgings during senior high school were particularly vulnerable to stress and internalizing problems compared to students living with their parents (Wannebo & Wichstrøm, 2010). Few studies have been published on adolescents moving from home to attend school at such an early age. However, studies among older adolescents, aged 19–20 years when leaving home to go college or university, show that students in lodgings struggle with homesickness, loneliness, depression and/or anxiety (Bayram & Bilgel, 2008; Blanco et al., 2008; Cleary, Walter, & Jackson, 2011; Stroebe, van Vliet, Hewstone, & Willis, 2002). A Norwegian study, exploring the meaning of living in lodgings during senior high school, illuminated a multidimensional transition among the lodgers, suggesting that they strive between contrasting experiences related to time, social life, independence development and self (Wannebo, Devik, & Uhrenfeldt, 2017). Hence, adolescents at the age of 15–18 living in lodgings may experience vulnerability regarding their own well-being and health throughout senior high school.

Improving and promoting health and well-being of populations is one of the main goals of the World Health Organization’s strategy, *Health 2020* (WHO, 2013). In the present study, we emphasize conditions that might be positive and helpful regarding adolescents’ mental health and well-being when living in lodgings during senior high school. Health and well-being are often viewed in relation to each other, and are phenomena with multiple meanings (Helsedirektoratet, 2015). In this study, mental health/well-being is understood as an experience of feeling well, living a good life, and being able to carry out smaller and bigger life projects (Dahlberg & Segesten, 2010).

Several studies have examined how adolescents experience mental health, and what the factors are that influence their well-being. In a Swedish study among adolescent girls aged 13–19, Larsson, Johansson Sundler, and Ekebergh (2012) found that togetherness, meaningful relations with others and balancing different aspects of life were important for the girls’ experience of health and well-being. In their everyday lives, balance, exemplified as stability in relationships with family and friends, was linked with feelings of well-being, while imbalance disrupted this state and called for action and change. According to Johansson, Brunnberg, and Eriksson (2007) and Landstedt (2010), adolescents perceive mental health as an emotional experience, described as “how you feel” in terms of self-esteem, stress and confidence. The emotional experiences are both positive and negative, such as feeling happy or unhappy. According to Johansson et al. (2007), the prerequisites for feeling well are relational, with family, friends and school being the three most important determinants of mental health. Influencing factors of adolescent mental health and well-being have been associated with circumstances of dynamic social processes, such as social interaction, performance and responsibility (Landstedt, 2010; Landstedt, Asplund, & Gillander Gådin, 2009). The social interaction process includes aspects such as good and supportive relationships with friends, family and teachers; respect; interaction in peer groups; risk of receiving disrespectful treatment; and assault. Performance consists of achievements related to school and leisure activities, as well as expectations about appearance and behaviour, having both positive and negative impacts on well-being. Responsibility can be illustrated as a continuum, ranging from a low degree of responsibility, exemplified as ignoring demands for which one is expected to take responsibility, to a burden, exemplified as demands related to achievements, performance and relations to others (Landstedt et al., 2009). Adolescents strive to obtain balance in these social processes, according to Lipworth, Hooker, and Carter (2011), who argue that health and well-being involve a state of balance in life.

In summary, previous studies indicate that adolescents living in lodgings during senior high school are vulnerable regarding mental health and well-being. Adolescents, in general, experience health and well-being to be an emotional experience in terms of self-esteem, stress and confidence. Influencing factors of well-being are associated with processes of social interaction, performance and responsibility, having both positive and negative impacts on well-being. Obtaining balance in these processes seems essential. However, there is little knowledge about what adolescents at the age of 15 to 16 living in lodgings during senior high school need to feel well, as they are in multidimensional transition. To promote their well-being, a more comprehensive understanding is needed of what the lodgers experience as crucial to feeling well and managing through this transition. Most of the adolescents living in lodgings seem to manage well, and listening to their experiences will be of great value. Knowledge about what the lodgers do and the conditions that help them when facing different challenges will provide a better understanding of how they can be supported and how their well-being can be promoted. Thus, the aim of this study is to explore what adolescents experience to be of importance and helpful for their well-being when living in lodgings during high school.

## Method

This qualitative study is based on both a phenomenological (Heidegger, 1996) and hermeneutical (Gadamer, 2004) understanding in the sense of searching for answers and truth through the lived experiences of the participants and interpretations by the researcher, a co-creation between the two. Meaning occurs through a circle of reflective writings and interpretation between the whole and parts of the text, and between pre-understanding and understanding (Alvesson & Sköldberg, 2008).

The study is performed by qualitative in-depth interviews, searching for descriptive experiences of the participants’ lifeworld (Kvale & Brinkmann, 2009) using an inductive qualitative content analysis approach. Content analysis is a method of analysing written or verbal communication in a systematic way, and it is useful in analyses of a person’s or group’s experiences, reflections and attitudes (Graneheim & Lundman, 2004). The method is used to inductively and systematically generate meaningful categories and concepts from data that describe the research phenomenon (Elo & Kyngäs, 2008; Graneheim & Lundman, 2004). Due to a longer intermission in the PhD study of the first author, interviews were performed in 2009 and in 2016.

### Participants and recruitment procedures

The qualitative research design demands limitation in the number of participants (Patton, 2002). Thus, we aimed at recruiting participants who lived in lodgings and represented a variety of adolescents regarding gender, age and study programme. Three senior high schools in rural central Norway (Nord-Trøndelag County) were contacted and asked for permission to carry out the study in 2009; two of the schools were asked again in 2016. Through the school counsellor, all the first and second year students living in lodgings were invited to a meeting where they were provided with additional information. Afterwards, they could sign up to participate in the study. More than 80% of the lodgers volunteered to participate. The inclusion criterion was that the students had at least one semester’s experience of living in lodgings. The students who consented to participate were invited to a meeting with the first author where they got more information and the opportunity to ask questions.

Twenty-one adolescents of both genders and from different study programmes were drawn to participate in the study, 11 during the winter of 2009 and 10 during the spring and autumn of 2016, respectively (Table I). The participants represented both first- and second-year students, and had between 6 and 20 months of experience as lodgers. Some of them moved from home at the age of 15, but all the participants were between 16 and 18 years of age at the time of the interviews. Eight of them lived alone as the only lodger in a private house, while 13 adolescents lived together with other lodgers. They all had separate bedrooms but shared a kitchen, bathroom and perhaps a living room with other lodgers.

**Table I.** Characteristics of the 21 participants.

Participants Girls, age	Living (lodgers)	Study programme	Participants Boys, age	Living (lodgers)	Study programme
“Hilde”, 18	Alone	Voc.edu.pr.	“Ola”, 18	Alone	Voc.edu.pr.
“Bente”, 18	Alone	GS*	“Arne”, 18	Together (3)	GS
“Silje”, 16	Together (1)	GS/Music	“Espen”, 18	Together (2)	GS/Music
“Lise”, 17	Together (1)	GS/Music	“Per”, 17	Together (2)	GS/Drama
“Mari”, 18	Together (1)	GS/Sports	“Gunnar”,	Together (3)	GS/Drama
“Vera”, 16	Alone	GS/Sports	17	Together (1)	GS
“Kristin”, 18	Together (2)	GS/Music	“Tom”, 16	Alone	GS/Music
“Anne”, 17	Alone	GS	“Erik”, 16	Together (1)	Voc.edu.pr.
“Mona”, 16	Together (2)	GS	“Frank”, 18	Alone	Voc.edu.pr.
“Clara”, 17	Together (3)	Voc.edu.pr.	“Yngve”, 17		
“Dordi”, 17	Together (1)	Voc.edu.pr.			
“Nina”, 17	Alone	Voc.edu.pr.			

*GS = General Studies

### Data collection

The study was carried out in the context of the senior high school, a context familiar to the first author as a former school nurse. Due to the participants’ ages, it was vital that they felt comfortable to tell their story (Kvale & Brinkmann, 2009). Hence, all interviews were conducted at times and locations suggested by the participants, most often in a pleasant room at the school or at the participants’ lodgings. The first author conducted all interviews, and began each session with a broad conversation before turning on the recorder. The interviewer was focused on the relation between the interviewer and the participants, being attentive to possible signs of unpleasant feelings among the participants as they were asked to tell about personal, and possibly difficult or challenging experiences (Sandelowski & Barroso, 2007). The relationship is dependent on the awareness of the interviewer, emerging through her communication (Rogers, 1956). Thus, the interviewer tried to be genuine in listening and encouraging, while simultaneously being aware of non-verbal signals and being sensitive towards the participants’ vulnerability.

An interview guide divided into four parts with open-ended questions shaped the interview. In the first part, the participants discussed their backgrounds and described the room(s) and their relationship to the host. Secondly, they told about their experiences living in lodgings. They were asked to describe what they usually do during an ordinary day, from getting up in the morning until falling asleep at night. The interviewer supported and encouraged the participants by nodding and providing positive comments such as “That was interesting; please tell me more.” When it was considered beneficial, follow-up questions were asked. Thirdly, there was a semi-structured section with questions about different aspects of living in lodgings, such as “What is important for you to do/manage well when living in lodgings?”, “What helps you, and gives you a feeling of succeeding and feeling comfortable?” and “Can you tell me or describe what you do when you face different kinds of difficulties and challenges?” Finally, the participants were given a future challenge. They were asked to imagine visiting a junior high school and telling the final-year students about their lives in lodgings. Questions such as “What would you tell them about living in lodgings?”, “How should they prepare themselves?”, “Any good advice you would give them?” and “Is there something they should be cautious about?” were asked. After the interview, the participants and the interviewer had a short conversation to reassure that the participants felt well and that the researcher was very grateful for their contribution. The same interview guide and procedures were used both in 2009 and in 2016. The interviews in both terms ranged from 48 to 90 minutes, with an average of 65 minutes. The interviews were audiotaped and transcribed verbatim.

### Data analysis

The interviews were analysed by means of qualitative content analysis as described by Graneheim and Lundman (2004) and Elo and Kyngäs (2008). The interviews were read through several times to obtain a sense of the whole. Next, text about the participants’ experience of important and helpful conditions and strategies for their coping and well-being when living in lodgings was extracted and brought together (approximately one-third of the transcribed text), which constituted the unit of analysis. The analysis was performed in several steps. First, a number of content areas were recognized. A content area is a rough structure of content that can be identified with little interpretation (Graneheim & Lundman, 2004); e.g., contact with teachers, parents, friends, self-care, coping, planning and prioritizing. Second, the text within each content area was divided into meaning units, and the meaning units were condensed and labelled with a code as close to the participants’ expressions as possible. Third, the codes were interpreted and compared for differences and similarities, and 36 tentative subcategories and 14 categories were abstracted, in a process involving a back-and-forth movement between the whole and the parts of the text (Graneheim & Lundman, 2004). Finally, through a process of reflection and discussion, the authors agreed on a set of two categories and six subcategories.

### Ethical considerations

The Regional Research Ethics Committee of Central Norway (REC central) approved the study in 2009 (REK: 4.2008.2240), and the Norwegian Social Science Data Services granted permission for research in 2016 (No. 49420). All participants were informed about the study both orally and in writing. They were asked to provide a written informed consent. Participants under the age of 18 received an information letter for their parents to sign. They were informed that it was possible to withdraw from the study at any time, and that all personal data would be anonymized and that they were guaranteed strict confidentiality.

## Findings

In exploring what adolescents living in lodgings during senior high school experience to be of importance for their well-being, two categories and six subcategories were identified: (1) Conditions for well-being, me and my surroundings: (a) practical support; (b) relational support; (c) convenient housing; and (d) supportive class environment. (2) Strategies for well-being, me and myself: (a) practical strategies, and (b) strategies facing challenges. The categories are described, and illuminated by quotations.

### Conditions for well-being—me and my surroundings

The first main category can be described as preconditions for well-being, a foundation in terms of positive transitions and what the adolescents experience they need from their surroundings to feel well, manage their lives and be able to carry out the “project” of living in lodgings. The category consists of four subcategories: “practical support”, “relational support”, “convenient housing” and “supportive class environment”.

#### Practical support

All the participants experienced different kinds of practical support to be of importance in managing their lives in lodgings. First, they all received some kind of help and advice from their parents. It may have been money to buy food or to pay the rent (if the scholarship was too small), leftovers and food supplies for the next week, having their clothes washed when home for the weekend, or a helping hand to fix things that were broken. Likewise, advice and supervision about housekeeping and economy, but also about possible problems regarding schoolwork, were highly appreciated and needed:

Silje (16): I always ask for advice. Mom about social occasions, but Dad is the one I use when it comes to school and things are difficult … Dad, what should I do first, Dad, what can I put off? Can you help me, what shall I do … I always have someone to ask for advice, and that feels good.

Second, the lodgers in general viewed knowledge and practical experience from home as positive and helpful. Some had learned how to cook and prepare meals, others had learned how to sort and wash clothes or to clean the house. This kind of knowledge and experience was regarded as valuable for their ability to manage life, indirectly contributing to feelings of well-being:

Per (17): My mom bakes a lot of cakes, and so do I. The girls don’t bake, but I very often bake. I learned from my mother, if it is wheat bread or if it’s cakes or whatever. So on birthdays I bake cakes, and on Sundays I perhaps make a brownie … and things that Alice and Laura [co-lodgers] have not tasted before, and we have a nice time.

Finally, practical help and adjustment at school were mentioned as important and helpful. One of the schools had a later start on Mondays, and this was appreciated by the lodgers. Starting at 10 o’clock, they could stay at home on Sunday evenings or get some extra sleep before a new week. A shorter school day in the middle of the week also helped. It meant something to look forward to, more time to prepare a hot meal, or just to relax and get some extra sleep. The teachers were also mentioned for their practical help. One lodger experienced that her teacher took into consideration that her living conditions were more demanding than the other students’. The teacher even offered practical help with her food supply and driving her to the doctor when she was sick:

Mona (16): One time I was … I had to go home, and then my teacher asked if I managed well, because she knew that I had anaemia. And then she said … she somehow took the responsibility for me and just bought food. She said “If there is anything you just have to tell me, and I will take care of practical tasks.” And then I was so glad, I almost started to cry.

#### Relational support

The lodgers experienced several kinds of interactions and support from their surroundings, including from parents, family, friends, schoolmates, teachers and school personnel. These relationships and support seemed crucial for the lodgers’ well-being. As they were struggling with different challenges, they experienced being seen and supported by their parents and teachers to be of great value. Engagement and care from teachers made the lodgers feel important and confirmed. Many participants spoke of the importance of having an adult nearby to talk to, as talking to an adult who cared and had some kind of competence was different from talking to a friend. Conversations were experienced as essential when facing challenges. The lodgers emphasized trust, security, availability and being listened to, in their relations with teachers and adults:

Dordi (16): My teacher … I think she is very clever. She sees you and … She knows me well because she was my teacher last year as well, so she can see if I have a hard time. Then she takes me aside and out of the classroom. She perceives it. I don’t have to say anything: she just sees that now Dordi doesn’t feel well. I like that she brings me out of the classroom. Then I feel that she only focuses on me. It is a great help for me.

Available adults and engaged teachers were important, both during problematic periods, and the first semester as the lodgers strived to settle into their new lives. Close contact and regular conversations with their class teacher during the first months, and attention from teachers when the lodgers were absent from school, was experienced as confirming somebody was engaged with them and cared about them. In contrast, some participants experienced that the teachers did not care or pay attention or understand their situation, and that made them feel bad. One lodger skipped school when she had problems, without receiving any reaction from her teacher. Tom expressed a lack of understanding and support:

Tom (16): Right now it’s much harder to go to school, to prepare for a test … and the teachers lack understanding about me having a hard time performing at school compared to others that gets … parents make food and parents tidy and parents do everything. There is nobody looking after me, nobody makes sure I feel mentally well, or when I am sick I have to manage on my own. To feel alone, nobody but you, knowing that you have to manage on your own, that can be very scary … so I just wish that the school could have a support person.

Most of the lodgers experienced that their parents were engaged and interested in their lives in lodgings. The parents were available and easy to contact. Some of the lodgers contacted their parents every day, just to talk and tell them about the day, while others made phone calls when they needed advice or just somebody to talk to. The interaction, care and support from family both showed confidence in their adolescents, and were crucial for the adolescent to feel well and able to manage their lives:

Lise (17): My dad has been magnificent. He makes it very clear that he wants me to come home, and he is looking forward to see me. But if I say I have other plans, then okay, “It’s a pity, but I am glad you are having fun”, and he doesn’t make a big deal out of it. So they see me becoming an adult and behaving like that, which really helps me. I feel good about it.

Some lodgers experienced they needed parents to take control, set limits and/or help them get back on track when something got out of control:

Vera (16): My parents said “If you don’t pull yourself together Vera, you have to move home again, and that will be tomorrow!” Actually, I am very glad they do such things … I think I could have managed the situation on my own as well, but sometimes parents just have to help, and that feels good.

Most participants found it important to be together with friends or family. It was about filling the leisure time, being socially active and not feeling lonely. It was about being included, being a part of something and having feelings of belonging, as the lodgers had been uprooted from their social environment of origin. Some of the adolescents found new friends or became members of a new group, meeting each other several times a week, or “meeting all the time” on social media. They spoke of this company and togetherness as “safe”, “meaning everything”, or even stronger, “helping me survive”:

Gunnar (17): My circle of friends are those working and hanging [sic] at the Internet café and those hanging out there. We play together or just talk. My circle of friends is the reason I survive … To be in contact with other people, it makes you feel that you are something. It’s very important.

Espen (18): I have a lot of good, creative people around me, and I feel it is a steady group, you know, safe. And that’s why it is fun meeting people, it’s valuable meeting people you can trust, without being afraid of being pushed away or being replaced. I feel I have a circle of friends here that is safe to a larger extent than earlier in life.

Others, in contrast, preferred the valuable contact with family members and frequent family gatherings at weekends:

Dordi (16): The whole family means a lot to me, now even more than before. Both grandparents, aunts and uncles, cousins and … yes. We are quite a large and close family and we meet on weekends.

Having friends and belonging means close relationships, understanding, sharing interests and receiving positive feedback, all important to feel well. For the lodgers, it also meant something to look forward to, helping them through the week, knowing that every Monday or another day they would meet friends for a meal or social activity:

Ola (18): We are a group of 7–8 boys who meet every Monday in my apartment to play Bingo. Then we drink coffee, and we bake Swiss rolls and cake with macaroon filling and such things. It’s just to have something to do on Monday evenings, we really look forward to this.

In contrast, some lodgers experienced being alone, not having close friends to trust or not being part of any social gathering. This is difficult—as expressed by Tom:

Tom (16): It has been difficult to be social and socializing because you have a new life when you live alone. You are used to family routines, but in lodgings you have to manage all by yourself. If you want to be social, you have to take that step, and that’s not easy. It affects you when being alone. I think a lot about this, being alone is quite unpleasant.

#### Convenient housing

Physical environmental conditions were emphasized by the adolescents to have an impact on their everyday lives in forming a basis for positive transitions. The rented room or flat was given much attention by most lodgers. Practical, tidy and nice rooms with ample space and necessary equipment seemed essential to feel well. Some lodgers started early to find “the right place” to live, looking for rooms with bright colours and space to accommodate all their belongings. Other participants moved from their first room as it felt unpleasant and affected their well-being:

Clara (17): The kitchen was so small, with a little sink in the corner. It was very gloomy with brown walls, ordinary old wood you know, it was not pleasant at all. So yes, I feel much better now because it is more open and bigger, and it seems so much easier, i.e., we have a washing machine. I feel much better now because last year it was … it seemed as I got weak because I almost didn’t eat because I almost could not prepare food. There were always a lot of dirty dishes around.

### Supportive class environment

Since the adolescents were leaving their familiar environment, the social environment at school and in classes was important for them to feel well. Most of the lodgers experienced a period of insecurity in the beginning, but little by little they got to know their classmates better, and found other students with mutual interests or they felt stimulated by the environment at school. A nice, friendly and stimulating environment in class and at school made the lodgers look forward to school and gave a feeling of well-being:

Kristin (18): When you move to a new place, you meet a lot of new people with whom you are together every day. It’s very important to know that you have people you like and you look forward to meeting at school. That is the most important thing because if you don’t feel comfortable at school, then you will not do well, and you will feel bad.

Espen (18): There are many nice students here at school, creative people … it takes a lot of effort for me to feel well. Good teachers … particularly in the art classes … many good teachers that want the best for us.

### Strategies for well-being—me and myself

The second main category concerns how the lodgers approach their new lives, and what they do themselves to feel well and to be able to carry out the “project” of living in lodgings. The category consists of two subcategories: “practical strategies” and “strategies facing challenges”.

#### Practical strategies

All participants spoke about different kinds of practical strategies that helped them get through various challenges. These strategies are consecutively presented under the headings “skills”, “routines and structure” and “creating a homey place to live”.

##### Skills

The lodgers experienced practical challenges that other adolescents of their age didn’t seem to need to think of. For instance, they had to take care of housekeeping, washing clothes and handling their finances as well as fix things that were broken. Most of the participants expressed the intention of developing their skills in order to be able to repair things themselves. The experience of repairing something, and the knowledge that they could handle both their finances and dirty laundry, gave the lodgers a feeling of satisfaction, important for their well-being:

Silje (16): I didn’t know how to wash my clothes, but I asked my Mom for help and I got a small introduction and tried on my own. This is how you learn step by step, and it becomes more fun … that feeling of coping is good.

Achieving results at school and/or feeling responsible and being able to take care of themselves also brought about a sense of well-being. Some participants worked hard to achieve results as this made them happy and satisfied. Others talked about adjusting their expectations to achieve their goals, or being aware of how even small steps improved their skills and developed independence and good feelings:

Espen (18): I went to the store and bought my first bag of groceries. Just that … the first bag, it felt good. To feel that you take responsibility, things that you haven’t done before. And it is practical too.

##### Routines and structure

All participants experienced life in lodgings to be busy at times, with different tasks and duties to fulfil. Feeling stressed and overwhelmed, most lodgers described strategies that involved planning, creating routines and structure, helping them to reduce stress, gain predictability and be in control. Some lodgers made time schedules or everyday routines like shopping on Mondays, washing clothes on Thursdays, eating dinner at half past four every day, etc. Others, living together with two or three lodgers, had plans for job-sharing like one week they were responsible for cleaning the kitchen and bathroom, or one week they were in charge of garbage sorting and vacuum cleaning. They described how establishing work habits had helped them to fulfil tasks and get work out of the way, promoting feelings of being in control and accomplishment. As there was a lot to think of during the week, some lodgers had developed recall strategies like reviewing the day every morning or making notes and lists. This helped them create a picture of what to remember and to organize daily life:

Vera (16): Every morning I try to think about what will happen during the day, what classes I have, and what I have to bring to school. Is there anything I have to remember today? I use to write notes or lists if there is something I have to remember or to do, I write a note and hang it on the mirror in the bathroom. Lists helps me when I write them, I get my thoughts sorted. Clearly, it helps me all the way.

Some prioritized withdrawing from social activities to make time for important duties like homework, and then relaxed on their own. Others, in contrast, prioritized to make time for friends and activities after school, as this made them feel well, and would rather stay up all night if they had urgent homework to do.

##### Creating a homey place to live

Most of the lodgers made an effort to keep their room(s) tidy and clean. When it was tidy, it was easier to concentrate, relax and feel well:

Hilde (18): I tidy and wash clothes, maybe three times a week. I like that it is tidy. I relax better in a chair when I have tidied and emptied the dishwasher … I am not house-proud in any way, but I feel well when it’s tidy around me.

Some participants made an effort to create a homey feeling, decorating their room to be a cosy, nice and homey place to live. Some brought things that held memories or things that had a special meaning to them. Others liked to give their room a personal touch or style with curtains, pictures and decorative items. Some lodgers also made an effort to create a homey atmosphere by lighting candles, or carrying on traditions from home:

Silje (16): I decorate for Christmas. I think it’s important in the time pre-Christmas, and I bring traditions and customs from home to my lodgings. I watch children’s TV every evening during Advent. I think it’s important to take care of the things that make me enjoy every day life and feel well, and the different seasons

#### Strategies facing challenges

The participants told about different strategies they used when facing various challenges while living in lodgings, strategies helping them to process unpleasant experiences and impressions, or promote feelings of well-being in general or when life was tough.

To “take care of myself” was an important issue after moving to lodgings, as the participants became aware of their responsibility to take care of themselves. They experienced two dimensions in this. First, they had to take care of their physical needs. Most participants spoke about the importance of proper food to feel well, gain energy and be able to manage all demands:

Kristin (18): I feel very well getting food when waking up in the morning, and I have bread and sandwich filling, and know that now I will not be hungry. Later I come home and then I prepare dinner. Actually it feels very good. I think lodgers who do not experience being short of food underestimate the importance of food. So food takes a lot of efforts for my well-being.

Some of the participants described strategies for taking care of themselves with regard to nutrition, like making their own bread, or routines of making dinner together with friends, one day each during the week. Others clearly were struggling, and found it hard to develop routines regarding meals:

Frank (17): I seldom have food in the cupboard, and the few times I make some food, then my room-mate eats it, and if he buys me something in return, he buys bread I don’t like. I am bad about food.

Second, “taking care of myself” was about being aware of their own needs in a broader sense, listening to their bodies and prioritizing what was good for them; e.g., when feeling stressed and exhausted, some participants decided to take a day off from school. Other lodgers described a strategy of allowing themselves to enjoy or indulge in something, as this gave them a good feeling and the necessary strength to go on:

Silje (16): I allow myself to enjoy eating something I want … it helps me a lot just to enjoy a piece of chocolate if I am tired of my homework … now I watch TV and enjoy myself. I haven’t been able to do that before, actually appreciate that I can do something nice for myself.

Lise (17): When I spend time just on my own … I get an extra good feeling when I have done something nice for myself, something that doesn’t lead to anything. Just doing something good for myself without any other causes, makes me feel very well.

Recreation and stimulation was another important issue for the lodgers. All participants had experienced the importance of both recreation and activity to feel well. As the life in lodgings was demanding, they all had developed strategies of relaxing, calming down and disconnecting from everyday busyness. Some found it hard to get enough rest and recreation, but they all talked about relaxation as crucial to managing their lives. Some had a routine of relaxing; e.g., for one or two hours after school every day, they lay on the sofa and watched TV. Others had at least one evening on their own in the middle of the week in which they created a cosy atmosphere with candles, enjoyed peace and quiet, read a book or watched a film. They used phrases like “disconnect thoughts”, “having time out” or using “cut-off elements”:

Gunnar (17): Occasionally I sit by the fireplace and I look into the flames. It’s just quite calm. I just sit in my own world … and then I have my cut-off elements where I either sit down and watch cartoons, or read comics, just completely disconnect, turning off social media and watch cartoons or read comics. That’s what I do; it’s important for me.

Involving themselves in activities or events were strategies described as stimulating, promoting positive feelings and energy, or feelings of being relaxed. None of the participants were engaged in sports or regular leisure activities, but many of them occasionally participated in physical activity, enjoyed walks alone or with friends in fresh air, or told stories about trips and positive experiences of nature making them feel well. Others enjoyed playing an instrument, going to concerts or participating in cultural events:

Vera (17): Music is very important. I play in my leisure time. It gives me a good feeling. It depends on the music, but music is all … there is nothing that makes me feel as successful as when I am standing on the stage, singing and playing guitar.

The lodgers also experienced the importance of balancing rest and activity to feel well. Some needed to be socially active and stimulated more than others, but most participants seemed to be aware of how they could best adjust rest and activity in relation to demands and tasks at school and everyday life:

Erik (16): Sometimes I decide to stay in town just to be alone … I want to be alone in lodgings all weekend, relax and be a slob and watch TV, because when I am home, I meet my mates, and we have a full programme. So if I need to relax and be lazy, I stay behind in lodgings and relax alone.

Handling feelings and thoughts was a third important issue for the lodgers. During their life in lodgings, all participants experienced situations or periods of conflict, difficult emotions and/or unpleasant thoughts. Some lodgers were struggling with emotions and thoughts, a kind of uneasiness and worry, and gradually they developed strategies to help them handle the flow of thoughts, and regulate or cope with their emotions. When feeling tense, sad or hurt, easing the pressure by crying, accepting and showing their feelings was of great help for some:

Tom (16): If I feel sad, I feed that sadness. Yes, then I just listen to sad music and I watch sad series and I watch sad films, and then I feel sorry for myself. It might seem strange, but in a way I feel it’s good to be in the sad emotions for a while … I don’t like ignoring my feelings and hiding them away.

Others tried to repress bad thoughts and feelings by finding something else to do, like watching TV, going to the movies, visiting friends or just not allowing themselves to think too much or be sad for too long. They felt that this helped as the thoughts and feelings disappeared or became less overwhelming, as opposed to ruminating and letting the thoughts become too troublesome. As an example, Vera used a special strategy to clear unpleasant thoughts from her head, a kind of self-cleansing strategy:

Vera (16): And I have another thing that’s very effective. If I feel that now, nothing matters and everything is terrible, then I go into the shower and just sit down, and I turn on very hot water. And then I just think that now I am nobody. It’s just … for a short while, please don’t think of anything right now, just be nobody. It’s very nice. It’s the only way I completely cut off.

The lodgers also described other ways of processing feelings and experiences. Some preferred talking to somebody, as described in the section on relational support. Talking helped the participants to organize and process overwhelming thoughts and feelings. However, the strategy used when experiencing complicated issues and thoughts involved a thorough assessment of what they would share and with whom:

Erik (16): In moments where you struggle with something as big as this, which is quite unpleasant to sit alone with, and none of your best friends know about it, then you consider somebody you trust 100% to talk to. Because I don’t know how I should have handled this. The moments this comes on to me … so I told Anders because I didn’t have any choice.

Even if talking to others was a preferred strategy, the lodgers experienced a need for balance within interactions and relationships with others to feel well. Some had experienced that close relationships to others could be too demanding if they shared problems with their friends, and vice versa. For example, the problems could be serious and the situation could be overwhelming and difficult to handle. Being there for each other entailed good feelings and affiliation. At the same time, they became aware of their own limitations and had to be selective about what to share with others, to protect their own vulnerability and well-being:

Espen (18): I think it’s important to talk. But at the same time … as I have experienced lately, I have to be careful about people who are in a similar situation as me, being busy, feeling stressed … Perhaps go to adults or parents for help instead.

Others preferred to withdraw, think and process on their own; e.g., alternately thinking and reading/watching TV or “living out” their feelings through PC games or role play:

Mari (18): I don’t speak much about difficult happenings. I prefer being alone then, because when I am alone, I sort of process what has happened. It’s not like I just lay there and think but, as an example, I watch TV, and then I can cut off and think about it for a while, and then I turn back to what I watch on the TV and so on.

Some described a strategy of writing down their thoughts, as this helped them to process thoughts and feelings, or just get them “out of the system”. Written words could take the form of a “self-instruction”, like nice words to keep the spirits up, or to help the lodger view things in perspective:

Lise (17): I have a book where I write down what is good about me, and what kind of person I want to be. When I read in the book I get some inspiration to keep on going after all. Actually, I think it’s smart to have such a book, just to turn back to when you feel hopeless, because you easily do that when living in lodgings … and just see that you are not so bad after all.

The lodgers also described how ways of thinking could help them handle difficult thoughts, feelings or conflicts. Some spoke of strategies to remove thoughts that were irritating or made them angry; e.g., use of irony or a process of ignoring and accepting the actions and habits of their co-lodgers. Others told how they reflected upon feelings, and tried to understand or find solutions to the problem or just ease the unpleasant feelings:

Silje (16): In the beginning I get a very pricking feeling. First, I get this lump in my stomach, and I sit with this feeling for quite a long while. But then I start to think, I have to get rid of this feeling, I can’t keep that … and then I start thinking of how I can save myself from this feeling, and it’s then I start thinking of what can be read between the lines. So, first I can have this pricking feeling, but then I think I have to change the feeling, and I do that by thinking.

## Discussion

This study aimed to explore what adolescents experience as important and helpful for their well-being when living in lodgings during senior high school. We identified several preconditions for well-being in the adolescents’ surroundings, which form a necessary basis for positive transitions. Further, the lodgers developed various strategies involving positive plans and actions to feel well. Our findings indicate that the lodgers developed knowledge and insight into their own well-being, both inward and outward, like “what I need and what I must do to feel well and be able to carry out the project of living in lodgings”. The preconditions identified were experienced as resources, but developing strategies were also required to activate and strengthen these resources. The lodgers built some of their strategies on the identified condition in order to achieve well-being. According to Meleis, Sawyer, Im, Messias, and Schumacher (2000), this knowledge and insight, resulting from the lodgers’ lived experience, are process indicators of a healthy transition; the lodgers developed new skills and strategies to manage the transition with regard to well-being (Meleis et al., 2000).

Available practical and relational support from both parents, family, teachers and friends were important preconditions for well-being. Practical support (Kendal, Keeley, & Callery, 2011; Malecki, Demaray, & D’Amato, 2003), as well as close relationships and daily communication (Hefner & Eisenberg, 2009; Offer, 2013), is found to be significant for adolescents’ emotional well-being. Knowing that they had engaged parents and teachers and/or close friends around made the lodgers feel safe, supported and confirmed, and it provided a sense of belonging. Modern technology and access to mobile phones made it easy for the lodgers to stay in contact with their family and friends. According to Meleis et al. (2000), the need to feel and stay connected is a prominent theme and dimension during transition. Making new contacts and continuing old connections, the lodgers utilized their social networks as important sources of information, confirmation and social support. Interactions with other lodgers, like making dinner together and visiting each other for activities and conversations, were strategies used to feel well, possibly indicating that the lodgers experienced an increase in their level of confidence (Meleis et al., 2000). Hence, our study illuminates that feeling connected is a dimension of transition as well as a precondition for positive transitions in terms of developing confidence and mastery of the skills and behaviours needed to manage their new situation as lodgers.

The outcomes provide a basis for illuminating and discussing the findings within the framework of the existential well-being theory of Galvin and Todres: “dwelling-mobility” (2011). According to their theory, experiences of well-being are particularly important to people as an inner resource when they are vulnerable and/or facing health-related challenges (Todres & Galvin, 2010). The theory of well-being as “dwelling-mobility” describes a capacity for movement and development as well as a capacity for settling and feeling at home. We discuss different kinds and levels of well-being in terms of both mobility/development, and dwelling, experienced spatially, temporally, interpersonally, bodily, in mood and in terms of the experience of personal identity (Galvin & Todres, 2011).

Practical support was an important precondition for well-being as were financial and practical help, advice and supervision from parents and teachers in helping the lodgers to manage different challenges. This support was experienced as a resource for feelings of safety, upon which the lodgers could build their practical strategies of developing skills and creating routines and structure. Developing skills and achieving results at school, as well as being responsible and able to take care of themselves, gave the lodgers a feeling of coping and satisfaction. This can be understood as the well-being experience of identity mobility: “I can” (Galvin & Todres, 2011). According to Galvin and Todres (2011, s. 8), there is a sense of “being able to”, a degree of confident personal competence in which one feels able to move into the future and achieve one’s values that are consistent with the knowledge of personal possibilities and self-beliefs. As the lodgers create routines and develop new skills, they might acknowledge their own strengths and potential, possibly giving them confidence and a feeling of being able to manage new challenges in the future.

Convenient housing was another important precondition for well-being, experienced as a resource for feeling comfort and at home, and inspired the lodgers to create a homey place to live. Keeping their room tidy, personal decorations, and creating a cosy atmosphere and appearance of their room, made the lodgers feel well, safe and relaxed. This can be understood as a well-being experience of spatial dwelling: “at-homeness” which Galvin and Todres (2011), emphasize as a sense of “being at home”. The lodgers were tuned into the spatial possibilities of their environment that offered settling or peace in ways that they valued or wanted. According to Galvin and Todres (2011, s. 3), having familiar objects and personal things close at hand connected the lodgers to their familiar sense of place and belonging, returning spatially to a sense of at-homeness as an experience of dwelling. Thus, physical surroundings that are comfortable and familiar are important for the lodgers to settle and feel well.

Further, relational support and a supportive class environment were important preconditions for well-being. Being seen, supported and confirmed, and experiencing togetherness, as well as being part of a friendly class environment and making new friends at school, were essential to the lodgers’ well-being. Several studies confirm the importance of relational support to adolescents’ well-being (Einberg, Lidell, & Clausson, 2015; Kostenius & Öhrling, 2006; Larsson et al., 2012; Larsson, Sundler, & Ekebergh, 2013). Relational support in our study was experienced as a resource for trust and security, care and support, confidence, confirmation and feelings of being included and belonging. Talking to friends, parents and teachers seemed essential in helping the lodgers to process difficult and overwhelming thoughts and experiences, or just being together to relax and feel well. This can be understood as a well-being experience of intersubjective dwelling: kinship and belonging (Galvin & Todres, 2011); the lodgers felt at home with another or others. A sense of familiar interpersonal connection constitutes relaxed situations of meeting in ways that made the adolescents feel they belonged there. In such situations of “kinship”, there is a sense of “we” rather than “I” and “you”; an effortless being together with one another, a sense of familiar security and togetherness (Galvin & Todres, 2011, s. 6). Thus, connecting lodgers might be important as they share the experience of living in lodgings, or facilitating connections between lodgers and other adolescents with shared interests, possibly finding an “at-homeness” with others. In addition, a stimulating environment in class made the lodgers look forward to school and inspired them to learn and develop. This might be a well-being experience of intersubjective dwelling-mobility: mutual complementarity (Galvin & Todres, 2011), an experience in which there are the qualities of stimulating interpersonal attraction as well as kinship and belonging. The mutuality is one of belonging; the complementarity is one of “a giving” in which one is more when together than when apart, a creative tension of “sameness” and “difference” (Galvin & Todres, 2011, s.6). In this creative tension, the well-being experience is like a journey of companionship that implies a feeling of being home with the other as well as “in adventure”. Thus, lodgers from different environmental backgrounds, sharing the experience of living on their own, could find both dwelling and development, if connected to each other.

“Taking care of myself” was one of the strategies used to promote well-being when living in lodgings. It was about being aware of and taking care of physical needs, as well as listening to their bodies, allowing themselves to enjoy something, and prioritizing what felt good for them. This can be understood as a well-being experience of embodied dwelling, to be in touch with one’s sense of “comfort” as a bodily experience (Galvin & Todres, 2011). According to Galvin and Todres (2011, s.10–11), one feels a sense of “being at home” in one’s body, a bodily comfort as a sense of familiarity and intimacy with the internal natural and organic rhythms of the body. Comfort constitutes a kind of well-being because it provides an embodied dwelling, a natural presence without other purposes than just being there.

Relaxing and disconnecting were important strategies in managing life in lodgings, regaining energy and feeling well. Enjoying peace and quiet, listening to calm music, lighting candles or sitting by the fireplace made the lodgers feel at rest and peace. Strategies when struggling with difficult emotions or unpleasant thoughts were mentioned as accepting and showing their feelings. “Living out the sadness” or emptying the head of thoughts by watching a film, or sitting in the shower with hot water flowing, being present in the moment, were examples of strategies helping the lodgers feel well. This can be understood as a well-being experience of temporal dwelling: present centredness (Galvin & Todres, 2011). When the lodgers were absorbed in the present moment of calm music, warmth and light from the fireplace, or hot water rinsing them, they were tuned into a kind of temporal focus that offers a sense of belonging or a deep connection with what is happening in the moment in ways that are wanted (Galvin & Todres, 2011, s. 4). In these moments, the lodgers are “brought home” to the event of just being, and there is a completeness and satisfaction in these moments of temporal dwelling, a sense of “being here”.

Stimulation and involvement in activities or positive events were strategies mentioned by some lodgers, providing energy and exciting feelings. This might represent the well-being experience of mood mobility: excitement or desire (Galvin & Todres, 2011). According to Galvin and Todres (2011, s. 7), this kind of well-being is a mood that has the quality of movement and buoyancy (zest for life), characterized by excitement. This energized feeling, exemplifying the experience of Vera, standing on the stage performing, constitutes the mood of motivation, a kind of vitality that sustains the feeling that life is worth living, a connection to her meaningful life desires.

The theory of dwelling-mobility was introduced as a conceptual framework to provide direction for caring and for studying well-being in different caring contexts (Galvin & Todres, 2011). To the best of our knowledge, making use of this theory has not been attempted when studying well-being in adolescents. Traditionally, adolescents’ well-being has been strongly related to several indicators of developmental trajectories; for example, engagement with school (Elmore & Huebner, 2010; Lewis, Huebner, Malone, & Valois, 2011), optimism and coping (Carver, Scheier, & Segerstrom, 2010) and resilience ability (Gilman & Huebner, 2006). Developmental elements were also among the findings in the present research, interpreted and reflected by the concept of mobility. However, elements of dwelling constituted the substructure for well-being in our participants and seemed to precede their well-being experienced in mobility. The feeling of homecoming or being home was emphasized, experienced spatially, temporally, interpersonally and bodily. Being uprooted from their parental homes and meeting different challenges, dwelling, finding comfort, and feeling safe and at home seemed more crucial for the adolescents’ well-being than development in the form of excitement and adventure. The interviews were performed during the first or second year in lodgings, and the adolescents needed time to adapt to the new situation. However, moving into lodgings opens a rapidly growing independence from parents, and the notions of being independent and responsible are expressed as very satisfactory by the lodgers (Wannebo et al., 2017), making the lodgers aware of their own development and abilities to manage on their own. Thus, as feelings of home are established, the well-being capacity of movement and development might be more important later on during the adolescents’ life in lodgings.

### Limitations and methodological considerations

Qualitative inquiry seeks to widen understanding, and generalization is not a goal. Our goal was to gain knowledge about young lodgers through achievement of an in-depth description of what the adolescents experience to be of importance and helpful for their well-being when living in lodgings. Data were collected at eight-year intervals, though in the same context. The sample consisted of participants of both genders, from varying study programmes and with different domicile origins, but none came from or moved to a city with more than 22,000 inhabitants. Some participants had been struggling throughout their life in lodgings, but none of them had serious problems or dropped out of school. With these limitations in mind, it is a strength that the sample size of 21 resulted in a rich and generous amount of qualitative research data, due to the participants’ eagerness to share their experiences. When working with a large amount of data, the success of content analysis depends on the coding process utilized. The large quantity of data was organized into smaller content areas in an effort to improve the trustworthiness and validity of the findings. The researchers spent considerable time identifying meaning units and a clear coding scheme, abstracting categories and subcategories several times, but the risk of overlooking potential key categories cannot be excluded. The method of qualitative content analysis, described by Graneheim and Lundman (2004) and Elo and Kyngäs (2008), offers a stepwise model presenting the analysis as transparently as possible. The authors reflected on and critically worked with the analysis until a consensus was reached. The trustworthiness of this study is linked to the stepwise planning and transparency in design and implementation.

## Conclusion and implications

Adolescents living in lodgings during senior high school experience several conditions present in their surroundings—such as convenient housing; a supportive class environment; and support from family, friends and school—to be of basic importance for their well-being. Building on these conditions, the lodgers found and developed individual strategies to feel well and promote their own well-being. Their experiences provide an insight into their lives, what they need from their surroundings, and what they do to manage the life in lodgings in terms of different kinds and levels of well-being. Our findings indicate that elements of dwelling might precede elements of mobility in the early stage of adolescents’ transition when moving away from the parental home. School nurses, teachers and other school personnel, aiming to promote well-being and prevent health problems among lodgers, should raise their awareness towards the preconditions for well-being identified, asking how they can support and contribute to the lodgers’ own strategies of promoting well-being, facilitating well-being experiences of dwelling and feeling at home. Interventions should be directed towards developing a positive and inclusive class environment as well as enhancing social support in terms of practical support, emotional support and development of new relationships. Availability and close contacts during the first semester should be emphasized, offering supportive conversations with class teachers and/or school nurses, focusing on how to strengthen each lodger’s individual strategies of well-being, as well as information about where and from whom they can seek support and help if needed. For further research, we recommend intervention studies, developing and implementing a follow-up programme focusing on well-being among lodgers in senior high school.
